# Motivational Climate and Physical Activity: A Multigroup Analysis in Romanian and Spanish University Students

**DOI:** 10.3390/ijerph16112013

**Published:** 2019-06-05

**Authors:** Félix Zurita-Ortega, Georgian Badicu, Ramón Chacón-Cuberos, Manuel Castro-Sánchez

**Affiliations:** 1 Department of Didactics of Musical, Plastic and Corporal Expression, University of Granada, 18071 Granada, Spain; manuelcs@ugr.es; 2 Department of Physical Education and Special Motility, Faculty of Physical Education and Mountain Sports, Transilvania University of Brasov, 500068 Brasov, Romania; georgian.badicu@unitbv.ro; 3 Department of Research Methods and Diagnosis in Education, University of Granada, 18071 Granada, Spain; rchacon@ugr.es

**Keywords:** family, motivation, physical activity, university students

## Abstract

Background: Motivational climate in sport is a psychosocial construct which is related with several factors, such as healthy habits and well-being, and is influenced by teachers, trainers, and parents. The aim of this study was to assess the relationships between motivational climate, family functionality, and physical activity within a population of students from Spain and Romania. Methods: A cross-sectional study was conducted on a sample of university students specialization: physical education (*n* = 605; 20.71 ± 2.42 years old), using the perceived motivational climate in sport questionnaire-2 (PMCSQ-2), the physical activity questionnaire for adolescents (PAQ-A), and the family functionality scale (APGAR) as the main instruments. IBM SPSS Amos was used for data analysis in the structural equation model that was developed. Results: We observed positive relationships between task-oriented climate, family functionality, and the level of physical activity, showing higher regression weights for Spanish university students. Ego-oriented climate was negatively related to family functionality in Spanish university students, while this association was positive in Romanian students. Moreover, the relationship between physical activity and functional family was stronger in respondents from Spain. Conclusions: It can be pointed out that a better family functionality can promote higher levels of physical activity and self-determined motivations in sports shown by task-oriented motivational climates. Thus, it is essential to take into account the influence of family in the promotion of healthy lifestyles.

## 1. Introduction

The university stage represents a critical period in the acquisition of behavioural habits, due to the fact that a large part of the students leave the family home to move to the place where they begin their university studies. Therefore, young people begin to live independently, separating themselves from the family nucleus [1]. Due to the characteristics of this stage, in which the individual is forced to meet their needs related to cleaning and feeding in the company of others, there are many studies that focus their analysis on the university population [2,3,4,5].

Although, in some cases, students leave the family nucleus due to their mobility to pursue higher education, this socializing agent still plays an important role in the lives of young people. This is because many students continue to live in the family home, maintaining the influence of it. The subject moves away from the family nucleus, prioritizing social relationships with their peer group in the adolescent stage [6]. Nevertheless, family relationships are improved after this stage due to the new role acquired by the young people and the need for support in economic, academic, and labour contexts. Therefore, family can influence the behavioural habits of young people, being able to favour both adaptive and maladaptive behaviors [7].

Due to the change that comes with the university stage, the practice of physical activity is considered a factor of vital importance regarding the development and maintenance of healthy habits and quality of life, as well as the decrease of harmful behaviors related to the consumption of harmful substances or the development of sedentary habits [8]. The practice of physical activity influences the level of general health, but also affects other variables, such as academic performance, self-perception, consumption of alcohol and tobacco, or social relationships. Thus, it is essential factor that affects the development of the individual [9,10,11].

The benefits of practicing physical activity are undoubted in the prevention and treatment of various chronic diseases such as obesity, hypertension, and diabetes, among others [12,13]. There are numerous studies that confirm these benefits [14,15,16] and give physical activity an important role in the control of morbidity and premature mortality caused by chronic noncommunicable diseases [17]. In fact, according to the WHO [18], chronic noncommunicable diseases will represent 75% of deaths in developed countries by 2020. Moreover, several studies have shown that the practice of physical activity has a multitude of cognitive benefits, such as the improvement of perceived well-being, increases in self-esteem and motivation towards healthy habits or a decrease in levels of stress and anxiety [19,20,21].

For this reason, it is of special interest to study the motivational factors associated with the practice of physical activity. There are many researches carried out in different contexts and populations that address this topic. For instance, Sevil et al. [22] established the differences between the levels of physical activities in secondary school and university students, while Chacón et al. [23] analyzed the importance of motivational climate in sport in the acquisition of healthy habits and the development of different psychosocial factors associated with cognitive and social well-being. Another study of interest is the one carried out by Diehl et al. [24], who found a close relationship between the practice of physical activity, motivation in young people, and the type of sport practiced.

In this sense, the achievement goals theory represents one of the main explanations for motivational factors related to sport [25]. This theory establishes that each person sets objectives in sports practice that will depend on the perception that one has of their own skills [25,26]. According to this theory, two types of motivational climates are developed in the contexts in which sports are practiced: the task-oriented climate and the ego-oriented climate. In the task-oriented motivational climate, intrinsic orientation goals are promoted, such as the effort to improve or the development of a leading role in sport or teamwork [27]. On the other hand, the ego climate is linked to extrinsic motivations, such as the pursuit of performance, competition, and social recognition [28]. In this way, it is interesting to study what motivational climates allow to promote physical-sport practice in a prolonged way and what are the mechanisms that generate such motivational climates, such as family, peer, and trainer influence or the socio-cultural context. 

Several studies have analyzed the existing relationships between the influence of family and the practice of sports in the countries where this study is carried out (Spain and Romania) [29,30]. These studies demonstrate how healthy habits and behaviour patterns, which are acquired in the earliest stages of life, are reproduced in the adult stage. In this respect, this study provides new information by analysing the influence of a structured family in the motivational climate in sports, in university students, as well as the level of physical activity, according to two clearly differentiated countries (Spain and Romania). 

For these reasons, the aim of this study was to assess the relationships between motivational climate, family functionality, and physical activity within a population of students from Spain and Romania using structural equation analysis with multigroup analysis.

## 2. Materials and Methods 

### 2.1. Subjects and Design

A nonexperimental, descriptive, and cross-sectional study was carried out on a sample of 651 university students from Romania and Spain that were enrolled in Physical Education courses, aged between 18 and 24 years (20.71 ± 2.42 years). The respondents participated voluntarily after receiving a detailed explanation of the objectives and nature of the study. Written informed consent was provided. We excluded from the analysis 46 participants that did not complete the inclusion criteria correctly (i.e., incomplete questionnaires, did not hand informed consent forms), thus the final sample was comprised of 605 subjects (368 males and 237 females): Romania (*n* = 178) and Spain (*n* = 427). The students from Romania were enrolled in the Transylvania University of Brasov, while the Spanish students were enrolled in the University of Granada. Finally, an assumed sampling error of 0.05 was taken into account, considering a random sampling by natural groups [31].

### 2.2. Instruments

The instruments employed in the research are shown. Given that the study sample is composed of Romanian and Spanish students, we proceeded to analyze the psychometric properties of all the instruments, observing good adjustment indexes (all of them are included in each instrument for both samples), as well as the internal consistency (Cronbach’s alpha). In addition, it is important to highlight that no instrument suffered changes in items or changes in its dimensions, since the validated versions of the scales were used.

The first instrument used is the Family Functionality Scale (APGAR), which comes from the original Family-APGAR version of Smilkein et al. [32], and adapted in Spanish by Bellón et al. [33]. This instrument is scored by a three-point Likert scale, where 0 is “Almost never”, 1 is “Sometimes” and 2 is “Almost always”. Finally, a summation was developed, allowing one to define three levels of family functionality. For Spanish university students, the scale showed the following values in the psychometric properties: KMO = 0.961, GFI = 0.953, AGFI = 0.949, CFI = 0.978, α = 0.88. For Romanian students, the values were: KMO = 0.947, GFI = 0.936, AGFI = 0.934, CFI = 0.955, α = 0.81. 

The second instrument was the perceived motivational climate in sport questionnaire (PMCSQ-2, [34,35]). This scale allows to establish two dimensions of motivational climate in sport through 33 items scored, using a five-point Likert-scale where 1 = Strongly Disagree and 5 = Strongly agree. This instrument comprised two factors, with three indicators each one. One factor is the task-oriented climate and its indicators are effort/improvement, cooperative learning, and important role. The second factor is the ego-oriented climate with the following indicators: punishment for mistakes, unequal recognition, and member rivalry. For Spanish university students, the scale showed the following values in the psychometric properties: KMO = 0.932, GFI = 0.986, AGFI = 0.984, CFI = 0.993, α = 0.85. For Romanian students, the values were: KMO = 0.922, GFI = 0.945, AGFI = 0.939, CFI = 0.956, α = 0.82. 

Finally, for older adolescents, we used the Physical Activity Questionnaire (PAQ-A), extracted from the original version developed by Kowalski et al. [36] and validated into Spanish by Martínez-Gómez et al. [37]. This scale is used in order to assess the level of physical activity performed during one week. The questionnaire allows to develop a summation of ten items, which are scored through a five-point Likert scale where 0 = Never and 4 = Always. For Spanish university students, the scale showed the following values in the psychometric properties: KMO = 0.941, GFI = 0.960, AGFI = 0.956, CFI = 0.969, α = 0.82. For Romanian students, the values were: KMO = 0.945, GFI = 0.966, AGFI = 0.962, CFI = 0.979, α = 0.84.

### 2.3. Procedure

First, we proceeded to request the approval of the study by the Ethics Committee of the University of Granada, which was granted with code "641/CEIH/2018.” Subsequently, the collaboration of different university centers was requested through an informative letter, elaborated by the Area of Corporal Expression of the University of Granada and the Area of Physical Education of the University of Transylvania. In addition, the informed consent of the respondents was requested through a document in which the nature of the study was detailed.

The data was collected during regular classes in the different university campuses. Different research assistants were present during the collection of data, in order to ensure that questionnaires were properly completed, to provide guidance on the completion of scales, and to answer questions. Moreover, participants did not receive incentives.

### 2.4. Statistical Analysis

**Figure 1:**
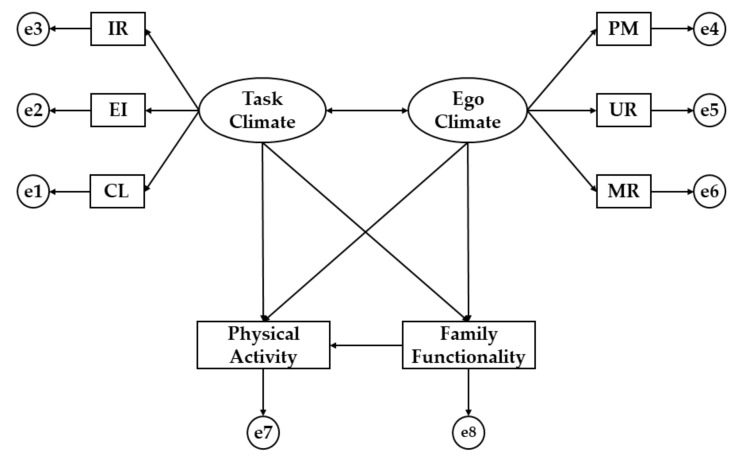
Theoretical Model. Note: IR, important role; EI, effort/improvement; CL, cooperative learning; PM, punishment for mistakes; UR, unequal recognition; MR, member rivalry.

The IBM AMOS® 23 (IBM Corp., Armonk, NY, USA) software was used to analyze the relationships between the involved constructs of the structural model. Once the theoretical model is developed, a path analysis is carried out considering the relationships of the matrix from a multigroup analysis, according to the country of the respondents due to the sociocultural differences of both (Romania and Spain). The path models consist of eight observable variables and two latent variables, each associated with three indicators (Figure 1). In these models, explanations of the associations between the latent variables are formulated from the observed relationships. Moreover, measurement errors (circles) are included in the observable variables so that they are directly controlled. The arrows are lines of relationship between the variables and these are interpreted as regression coefficients. The maximum likelihood method is used because it is coherent and invariant at the scale type.

The structural equation model (SEM) shown in Figure 1 is composed by two latent variables (ovals) and eight observed variables (squares). Task climate and ego climate are the latent and exogenous variables. These two exogenous variables were inferred by the following six observed variables: Important role (RI), cooperative learning (CL), and effort/improvement (EI) for task climate, and member rivalry (MR), unequal recognition (UR), and punishment for mistakes (PM) for ego climate. Other latent variables were physical activity and family functionality, which are related to task climate and ego climate.

## 3. Results

The path model showed correct fit indices in the parameters analyzed. *P*-value reveals a statistically significant value (*χ*2 = 116.864, df = 47, *p* < 0.001). This index should not be interpreted in a standardized way due to its sensitivity to the sample size. In this way, other fit indices were included, as established by Marsh [38]. The normalized fit index (NFI) revealed an acceptable value of 0.91, while the comparative fit index (CFI) and the increment fit index (IFI) showed excellent values of 0.95 for both parameters. Moreover, an acceptable value of 0.06 was obtained for the root mean square error of approximation (RMSEA).

**Figure 2:**
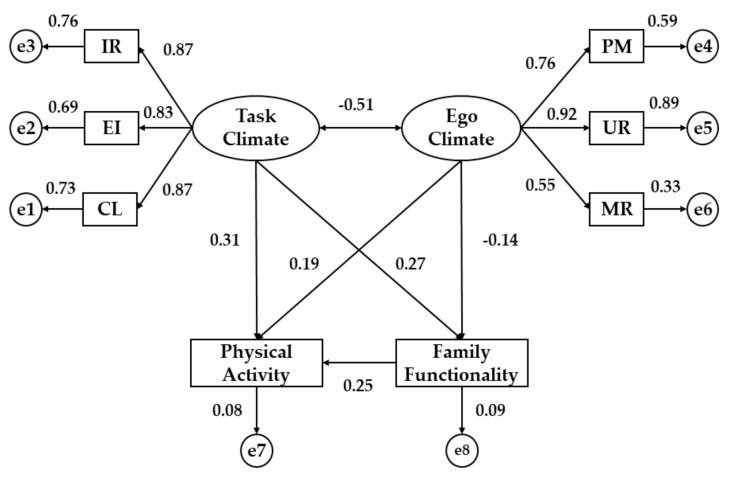
Structural Equation Model in Spanish university students. Note: IR, important role; EI, effort/improvement; CL, cooperative learning; PM, punishment for mistakes; UR, unequal recognition; MR, member rivalry.

First, the structural equation model (SEM) for university students from Spain (Figure 2 and Table 1) is presented. Statistically significant differences were obtained at the *p* < 0.001 level in the associations given between the two dimensions of motivational climate and its indicators. In this sense, the indicators that have the greatest influence on task climate (TC) were cooperative learning (CL) and important role (RI) to the same extent (*b* = 0.87), while the indicator that obtained the lowest regression weight was the effort/improvement (EI) (*b* = 0.83). For ego-oriented climate (EC), the highest association was shown by the unequal recognition (UR) (*b* = 0.92), while the indicator with the least influence was the members rivalry (MR) (*b* = 0.55). Moreover, the TC and EC were inversely related (*b* = −0.51, *p* < 0.001).

Regarding the relationship between motivational climate and the observable variables, physical activity (PA) was associated with task climate (*p* < 0.001; *b* = 0.31) and ego climate (*p* < 0.01; *b* = 0.19), in Spanish university students, showing a positive relationship in both cases. Moreover, functional family (FF) was directly associated with task climate (TC) (*b* = 0.27, p < 0.001), while this variable showed a negative relationship with ego climate (EC) (*b* = −0.14, *p* < 0.05). Finally, physical activity (PA) and functional family (FF) were positively associated (*b* = 0.25, *p* < 0.01).

**Figure 3:**
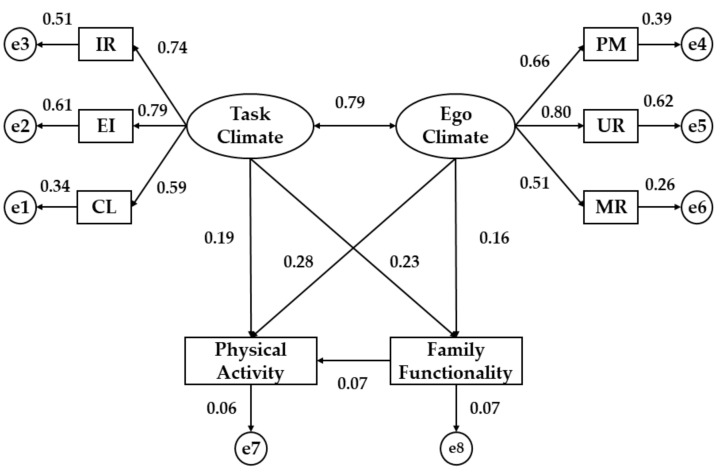
Structural Equation Model in Romanian university students. Note: IR, important role; EI, effort/improvement; CL, cooperative learning; PM, punishment for mistakes; UR, unequal recognition; MR, member rivalry.

Second, the structural equation model (SEM) for university students from Romania (Figure 3 and Table 2) is presented. Statistically significant differences were obtained at the *p* < 0.001 level in the associations given between the two dimensions of motivational climate and its indicators. In this sense, the indicator that has the greatest influence on task climate (TC) was effort/improvement (EI) (*b* = 0.79), while the indicator that obtained the lowest regression weight was cooperative learning (CL) (*b* = 0.59). For ego climate (EC), the highest association was shown by the unequal recognition (UR) (*b* = 0.80), while the indicator with the least influence was the members rivalry (MR) (*b* = 0.51). Moreover, the TC and EC were positively related (*b* = 0.79, *p* < 0.001).

When it comes to the relationship between motivational climate and the observable variables, physical activity (PA) was associated with task climate (*p* < 0.01; *b* = 0.19) and ego climate (p < 0.001; b = 0.28) in Romanian university students, showing a positive relationship in both cases. Moreover, functional family (FF) was directly associated with task climate (TC) (*b* = 0.23, *p* < 0.01) and ego climate (EC) (*b* = 0.16, *p* < 0.05), showing a low regression weight in the second case. Finally, physical activity (PA) and functional family (FF) did not show statistical associations (*p* = 0.127).

## 4. Discussion

Motivation in the practice of physical activity is a concept that has been studied worldwide as it is a fundamental issue nowadays [39,40,41,42,43]. After analyzing the results obtained, an inverse relationship between the task and the ego climate is observed in Spanish students, showing that an increased involvement in the task climate is associated with a decreased involvement in the ego-oriented climate. This situation is favored by the characteristics of Spanish society, that lead to taking greater pleasure and having more satisfaction in carrying out activities focused on hedonism and socialization, where a more relevant role is given to the involvement in the task climate rather than to the result achieved in the practice of sports [34,43].

On this basis, these circumstances can be created by family influence, since family provides different tools, habits, and motivations during childhood and adolescence, in order for children to perform physical activity in a more habitual way [44,45,46,47]. Another element that affects this situation is the climatic component, which is softer in Spain and allows numerous outdoor activities, such as those developed in natural environments like hiking or running, which are not as competitive as others and where family values can be promoted [48].

On the other hand, Romanian students show a positive relationship between task-oriented climate and ego-oriented climate, and that is because those university students who practice are motivated by the implication of task related indicators such as cooperative learning or effort to improve. In this sense, it can be established that climatic conditions can affect this situation, since the climate in Spain involves harsher autumns and winters, leading to sports activities being undertaken in closed spaces and competition activities being more common [49,50].

The indicator that has the greatest influence on task-oriented climate in Spanish students was cooperative learning. As it has been highlighted, cooperation in sports activities is essential, and it is one of the characteristics of athletes who are more focused on achieving a good team climate [51,52].

On the other hand, the capacity of effort and improvement was the category that scored higher in Romanian university students. These findings seem reasonable considering the most popular sports in this country, such as “oina”, handball, gymnastics, volleyball, or rowing. All these sports are practiced in sport halls, with a less hedonistic component and a clear competitive component associated with performance [50].

Nevertheless, the indicator that had the greatest influence on the ego climate was unequal recognition, something that is shown in both Romanian and Spanish students. This is due to one of the characteristics inherent of the athlete, which consists of the feeling of utility and the enhancement of sports performance by their own teammates and different agents of the sport context, such as fans or coaches. In addition, several studies showed how the ego climate is associated with competitive sports elements [24,51,52,53].

As it has been shown in Spanish students, the task-oriented climate is positively related to the practice of physical activity, influencing more than the ego-oriented climate. In this sense, it should be noted that sports policies adopted in Spain have been successful in the promotion of sporting habits as a way of life, giving greater value to activities that are oriented towards pleasure, leisure and a healthy lifestyle [53,54,55]. All this is further enhanced by the type of sport practiced in the Spanish region and the climate that normally exists in this country.

It should be noted that obesity levels are higher in Spain than in the rest of Europe [56], due to high levels of sedentary habits determined by digital environments [8,41,57,58]. This situation causes an increase of the prevalence of being overweight among young people and of pathologies associated with it. These pathologies are linked to physical and cognitive problems. In this regard, educational centers are developing intervention programs aimed at carrying out sports activities on a regular basis (at least three days a week), following the recommendations indicated by various entities [59]. In this regard, family plays a central role, since, as a socializing agent, it will help to create healthy habits during childhood that will become part of daily routine later in life, in adolescence, and early adulthood. On this subject, the configuration of intrinsic motivations linked to task-oriented motivational climates and the detriment of ego-oriented motivational climates is essential [60,61]. On the other hand, we can observe quite the opposite in Romanian students, because for them, the practice of physical activity is more linked to extrinsic motivations and ego-oriented motivational climates. That explains goals rather focused on results, performance, and comparison between athletes [62].

The task-oriented climate is positively associated with family functionality in Spanish students, while the ego climate is associated negatively. This data confirms that the practice of sport is largely associated with cooperative and collaborative practices, aspects that are linked to peer groups and family context. Some studies have confirmed this data, establishing a clear link between parents practicing physical activity on a regular basis and their children, who are also physically active. Therefore, this shows a close relationship between participation in and learning of sports through affective responses and behaviors prior to performance [60,61,62].

Therefore, the influence of family is a determining factor in the configuration of adaptive habits and behaviors of the emerging adult [63]. In Spain, the task climate is encouraged to the detriment of the ego climate, and outdoor activities, as well as those that involve the whole family are very popular [46,64]. On the contrary, in other countries of Europe, the climatic conditions do not enable and do not encourage outdoor sports. Finally, it should be noted that the practice of physical activity is positively related to family functionality in Spanish students, highlighting the findings of great value in the university context, about the two dimensions of the motivational climate in sport.

The limitations found in this study are mainly determined by developing a cross-sectional study, where data is collected at a certain time. Another limitation is that the data cannot be generalized to the general European population, since only Romanian and Spanish students have been analyzed. In addition, the total number of respondents analyzed in each of these countries is not representative for the total number of university students in Spain and Romania. Another limitation lies in the possibility of using an instrument focused on the families of students, in order to check their influence on behavioral aspects. On the other hand, the lesser influence exerted by family in the final stage of adolescence and in emerging adulthood represents an intrinsic limitation of the study. Finally, it is interesting to highlight the most important limitation presented by this research, which is associated with the instruments used for data collection. The Spanish students completed the scales adapted to Spanish. Nevertheless, the scales were not translated for Romanian students, so they had to complete them in English which could reduce the reliability and validity of the scales. However, it should be noted that for both samples, we obtained fit indices with acceptable psychometric properties (KMO, GFI, AGFI, …) as well as adequate values of internal consistency determined through Cronbach’s alpha coefficient, which shows that the data collected are suitable for the aim of the study.

## 5. Conclusions

The main conclusions of this research consist of a positive relationship between task-oriented climate, family functionality, and level of physical activity, showing higher regression weights for Spanish university students. On the other hand, ego-oriented climate was negatively related to family functionality in Spanish university students, while this association was positive in Romanian students. Furthermore, the relationship between physical activity and functional family was stronger in respondents from Spain. Thus, it can be pointed out that a better family functionality can promote higher levels of physical activity and self-determined motivations in sports, shown by task-oriented motivational climates. Therefore, it is essential to take into account the influence of family in the promotion of healthy lifestyles.

## Figures and Tables

**Table 1:** Regression weights for university students from Spain.

Relationship between Variables			Regression Weights				S.R.W
			Estimate	S.E.	C.R.	*P*	Estimate
FF	←	PA	1.935	0.633	3.998	**	0.25
PA	←	TC	2.564	0.629	5.237	***	0.31
PA	←	EC	1.741	0.648	3.519	**	0.19
FF	←	TC	0.845	0.588	4.367	**	0.27
FF	←	EC	−0.615	0.354	−2.159	*	−0.14
CL	←	TC	1.000	-	-	***	0.87
EI	←	TC	0.854	0.093	19.361	***	0.83
IR	←	TC	1.021	0.074	20.116	***	0.87
PM	←	EC	1.000	-	-	***	0.76
UR	←	EC	1.328	0.079	15.874	***	0.92
MR	←	EC	0.863	0.123	11.827	***	0.55
EC	↔	TC	−0.127	0.038	−7.411	***	−0.51

Note: S.R.W., standardized regression weights; S.E., estimation of error; C.R., critical ratio; TC, task climate; EC, ego climate; CL, cooperative learning; EI, effort/improvement; IR, important role; PM, punishment for mistakes; UR, unequal recognition; MR, member rivalry; FF, family functionality; PA, physical activity. * *p* < 0.05; ** *p* < 0.01; *** *p* < 0.001

**Table 2:** Regression weights for university students from Romania.

Relationship between Variables			Regression Weights				S.R.W
			Estimate	S.E.	C.R.	P	Estimate
FF	←	PA	0.432	1.241	0.757	0.127	0.07
PA	←	TC	0.812	0.377	3.411	**	0.19
PA	←	EC	0.796	0.583	4.991	***	0.28
FF	←	TC	0.545	0.416	3.852	**	0.23
FF	←	EC	0.764	0.659	2.358	*	0.16
CL	←	TC	1.000	-	-	***	0.59
EI	←	TC	1.005	0.099	16.231	***	0.79
IR	←	TC	0.936	0.128	15.637	***	0.74
PM	←	EC	1.000	-	-	***	0.66
UR	←	EC	1.419	0.063	17.746	***	0.80
MR	←	EC	0.888	0.149	11.627	***	0.51
EC	↔	TC	0.077	0.026	9.851	***	0.79

Note: S.R.W., standardized regression weights; S.E., estimation of error; C.R., critical ratio; TC, task climate; EC, ego climate; CL, cooperative learning; EI, effort/improvement; IR, important role; PM, punishment for mistakes; UR, unequal recognition; MR, member rivalry; FF, family functionality; PA, physical activity. Note 3: * *p* < 0.05; ** *p* < 0.01; *** *p* < 0.001.
